# Racial/Ethnic and Socioeconomic Disparities in Health-Related Quality of Life Among People With Coronary Heart Disease, 2007

**Published:** 2011-06-15

**Authors:** Donald K. Hayes, Kurt J. Greenlund, Clark H. Denny, Janet B. Croft, Nora L. Keenan, Jonathan R. Neyer

**Affiliations:** Centers for Disease Control and Prevention, National Center for Chronic Disease Prevention and Health Promotion; Centers for Disease Control and Prevention, Atlanta, Georgia; Centers for Disease Control and Prevention, Atlanta, Georgia; Centers for Disease Control and Prevention, Atlanta, Georgia; Centers for Disease Control and Prevention, Atlanta, Georgia; University of California at Los Angeles, Los Angeles, California

## Abstract

**Introduction:**

Health-related quality of life (HRQOL) refers to a person's or group's perceived physical and mental health over time. Coronary heart disease (CHD) affects HRQOL and likely varies among groups. This study examined disparities in HRQOL among adults with self-reported CHD.

**Methods:**

We examined disparities in HRQOL by using the unhealthy days measurements among adults who self-reported CHD in the 2007 Behavioral Risk Factor Surveillance System state-based telephone survey. CHD was based on self-reported medical history of heart attack, angina, or coronary heart disease. We assessed differences in fair/poor health status, 14 or more physically unhealthy days, 14 or more mentally unhealthy days, 14 or more total unhealthy days (total of physically and mentally unhealthy days), and 14 or more activity-limited days. Multivariate logistic regression models included age, race/ethnicity, sex, education, annual household income, household size, and health insurance coverage.

**Results:**

Of the population surveyed, 35,378 (6.1%) self-reported CHD. Compared with non-Hispanic whites, Native Americans were more likely to report fair/poor health status (adjusted odds ratio [AOR], 1.7), 14 or more total unhealthy days (AOR, 1.6), 14 or more physically unhealthy days (AOR, 1.7), and 14 or more activity-limited days (AOR, 1.9). Hispanics were more likely than non-Hispanic whites to report fair/poor health status (AOR, 1.5) and less likely to report 14 or more activity-limited days (AOR, 0.5), and Asians were less likely to report 14 or more activity-limited days (AOR, 0.2). Non-Hispanic blacks did not differ in unhealthy days measurements from non-Hispanic whites. The proportion reporting 14 or more total unhealthy days increased with increasing age, was higher among women than men, and was lower with increasing levels of education and income.

**Conclusion:**

There are sex, racial/ethnic, and socioeconomic disparities in HRQOL among people with CHD. Tailoring interventions to people who have both with CHD and poor HRQOL may assist in the overall management of CHD.

## Introduction

Coronary heart disease (CHD) is the number 1 cause of death among American men and women, causes 1 of every 5 deaths in the United States, and accounted for an estimated $177 billion in direct and indirect costs in 2010 (1). New approaches are needed to improve primary prevention, early detection, and clinical management of CHD. CHD and its risk factors have debilitating physical and mental effects on quality of life. Health-related quality of life (HRQOL) refers to a person's or group's perceived physical and mental health over time (2). HRQOL includes aspects of health such as physical functioning, social and role functioning, mental health, and general health perceptions that people experience directly. HRQOL is an increasingly important outcome in the study of disease because it reflects functional capacity, dependence, and productivity issues. HRQOL could affect adherence and compliance with treatment. Some studies have demonstrated that assessing changes in HRQOL could be a useful complement to clinical management of CHD by assisting in monitoring disease severity and progression (3-6).

Studies have documented less favorable HRQOL measurements in people with chronic disease compared with those without chronic disease including CHD (7-9). HRQOL may even identify people at increased risk for developing disease, as those with more risk factors for CHD report worse HRQOL than do those with fewer risk factors (10). Among people with CHD, those reporting less favorable HRQOL are women, Hispanics, people with depression and anxiety, single people, and people with higher severity of CHD (3,5,11). Few studies describe disparities in HRQOL among people with CHD in population-based data sets (7,9). The focus of our analysis was to identify socioeconomic disparities in 5 HRQOL measurements among community-dwelling adults with self-reported CHD using a national data set.

## Methods

The Behavioral Risk Factor Surveillance System (BRFSS) is a state-based, random-digit–dialed telephone survey of the US noninstitutionalized, civilian population. We analyzed the self-reported data from 427,269 adults aged 18 years or older in 2007 from the 50 states, the District of Columbia, Guam, Puerto Rico, and the US Virgin Islands. The median response rate among geographic units, based on the Council of American Survey and Research Organizations guidelines, was 47.8% (range, 26.9% in New Jersey to 79.9% in Guam). This rate reflects both telephone sampling efficiency and the degree of participation among eligible respondents contacted. The median cooperation rate for the 2007 BRFSS survey was 73.3% (range, 49.6% in New Jersey to 95.0% in Guam) and reflects the proportion of eligible people contacted who completed an interview. Additional details on the survey can be found at www.cdc.gov/brfss.

The HRQOL module has been used in BRFSS since 1993 and allows the assessment of general health, recent physical or mental health or both, and activity limitations (2). Participants provide subjective ratings of general health ("Would you say that in general your health is excellent, very good, good, fair, or poor?"), recent physical health ("Now thinking about your physical health, which includes physical illness and injury, for how many days during the past 30 days was your physical health not good?"), recent mental health ("Now thinking about your mental health, which includes stress, depression, and problems with emotions, for how many days . . . mental health not good?"), and activity limitations ("For how many days did poor physical or mental health keep you from doing your usual activities, such as self-care, work, or recreation?"). The questions have been validated with the medical outcomes short study form (12).

We analyzed 5 unfavorable HRQOL measurements among people with self-reported CHD, which we refer to as "unhealthy days measurements" when discussing them as a group. General health status was dichotomized as good/excellent (respondents reporting excellent, very good, or good health) or fair/poor. The number of days in the past 30 days in which a person reported constraints related to physical, mental, total (physical and mental), and activity-limited days was calculated as 14 or more days compared with less than 14 days. These unhealthy days measurements are traditionally used with BRFSS data, have been associated with chronic disease, and indicate a substantial level of impairment (13,14). In this study, we defined people with CHD as those who reported ever being told by a doctor or other health professional that they had had a "heart attack, also called a myocardial infarction," or "angina or coronary heart disease" during their lifetime.

Differences in the prevalence of each unhealthy days measure were assessed by age group (18-34, 35-49, 50-64, and ≥65 y), sex, race/ethnicity, and other socioeconomic indicators (education, health insurance coverage, annual household income, and household size). Self-identified race/ethnicity was either non-Hispanic white, non-Hispanic black, Hispanic (any race), Asian, Native American, or other. Native American was used for respondents who self-identified as being of American Indian or Alaska Native race. The "other" race category included respondents who self-identified as being of Native Hawaiian, Pacific Islander, or other, and those who indicated more than 1 race. Education levels were based on highest grade or year of school completed and categorized as not completing high school (<12 y), completing high school or its equivalent (12 y), some college course work, or college graduate or more. Respondents were considered to have health insurance coverage if they reported any type of health insurance. Annual household income was categorized as less than $20,000, $20,000 to $34,999, $35,000 to $49,999, or $50,000 or more, and as unknown/refused. Household size was categorized as living alone, with 1 other person, or with 2 or more people.

We excluded from our analysis observations with missing data on any of the unhealthy days measurements (4.2%) or CHD status (<0.1%), and we excluded pregnant women (0.8%), resulting in a sample size of 405,641; we focused the analysis on the 35,378 participants with self-reported CHD. Prevalence and 95% confidence intervals (CIs) of these unhealthy days measurements were determined for selected socioeconomic characteristics. Prevalence estimates were age-standardized to the 2000 US standard population except for those associated with specific age groups. Multivariate logistic regression models were developed for each of the 5 unhealthy days measurements; age group, sex, race/ethnicity, education, income, household size, and health insurance coverage were covariates. All covariates were entered into each of the 5 models to allow for comparison between the models. Data were weighted to reflect each state's noninstitutionalized, adult population.

Significant differences for estimates by characteristics for the 5 unhealthy days measurements were assessed by pairwise comparison tests with a reference group we selected for comparison. For the multivariate logistic regression model, reported *P* values for the *t* test of the beta coefficients are reported. A *P* value of <.05 was considered significant for the estimates by characteristics and in the multivariate logistic regression models. SAS version 9.2 (SAS Institute, Inc, Cary, North Carolina) and SUDAAN version 10.0 (RTI International, Research Triangle Park, North Carolina) statistical software were used to account for the complex sampling design so that accurate variance estimates could be calculated.

## Results

The overall prevalence of self-reported CHD in 2007 was 6.1%, increased markedly with age, was higher in men than women, was highest in Native Americans, and was lowest in Asians (Table 1). The prevalence of self-reported CHD decreased at higher levels of education and income. Respondents reporting less than 12 years of education and less than $20,000 of income had the highest estimates in their respective groups. The prevalence of self-reported CHD did not differ with household size or health insurance coverage.

Overall, respondents with self-reported CHD had worse HRQOL Tables (2a and 2b) than those without: 46.9% with CHD reported fair/poor health compared with 13.9% (95% CI, 13.6-14.1) of those without CHD; 41.0% with CHD reported 14 or more total unhealthy days compared with 16.4% (95% CI, 16.2-16.7) of those without CHD; and 20.9% with CHD reported 14 or more activity-limited days compared with 9.4% (95% CI, 9.2-9.6) of those without CHD (data for those without CHD not shown in tables). Among people with self-reported CHD, those aged 18 to 34 years had the lowest prevalence of fair/poor health, 14 or more physically unhealthy days, and 14 or more activity-limited days (Tables 2a and 2b). People aged 65 years or older reported the lowest prevalence of 14 or more total unhealthy days and 14 or more mentally unhealthy days.

In multivariable analyses (Tables 3a and 3b), adjusted odds ratios (AORs) for age groups 35 to 49 years and 50 to 64 years compared with those aged 18 to 34 years were 3.2 and 4.2, respectively, for fair/poor health status; 2.5 and 2.2, respectively, for 14 or more total unhealthy days; and 2.9 and 2.7, respectively, for 14 or more activity-limited days. Compared with people aged 18 to 34 years, people aged 65 years or older were significantly more likely to report fair/poor health status (AOR, 3.1) and 14 or more physically unhealthy days (AOR, 2.3); they were also significantly less likely to report 14 or more mentally unhealthy days (AOR, 0.4).

Women with self-reported CHD had similar prevalence estimates for unhealthy days measurements compared with men (Tables 2a and 2b). In multivariate analyses (Tables 3a and 3b), women had higher AORs for all unhealthy days measurements except for fair/poor health status compared with men.

All unhealthy days measurements were highest among Native Americans and lowest among Asians (Tables 2a and 2b). In the AORs, compared with non-Hispanic whites, Native Americans were more likely to report fair/poor health status, 14 or more total unhealthy days, 14 or more physically unhealthy days, and 14 or more activity-limited days (Tables 3a and 3b). Hispanics were more likely than any other racial/ethnic group to report fair/poor health status and less likely to report 14 or more activity-limited days, and Asians were less likely to report 14 or more activity-limited days.

There was an inverse relationship of unhealthy days prevalence estimates with education and income levels (Tables 2a and 2b), and the differences persisted in the adjusted analyses (Tables 3a and 3b). There were no significant differences in the prevalence estimates of unhealthy days with respect to health care coverage and household size except for a fair/poor health status and total unhealthy days for health coverage, and activity limited days for household size (Tables 2a and 2b). In the adjusted analyses, there were no significant differences with respect to health insurance coverage (Tables 3a and 3b); also, people living in households of 1 person were less likely to report fair/poor health status and less likely to report 14 or more activity-limited days compared with those living in a household of 3 or more people.

## Discussion

CHD prevalence varies across socioeconomic groups (12,15), and our study demonstrates variation in HRQOL among people with self-reported CHD across similar groups. Our study confirmed similar patterns shown in a study using data from the Medical Expenditure Panel Survey that identified impairment of HRQOL among people with CHD across age, sex, racial/ethnic, and income groups (9).

In our study, Native Americans and non-Hispanic blacks generally reported the highest number of unhealthy days, and Asians reported the lowest. Multivariable adjustment for age, sex, education, income, household size, and health care coverage suggested that many of the initial differences were accounted for by these confounders, particularly for differences between non-Hispanic blacks, Hispanics, and Asians compared with non-Hispanic whites. However, significant differences between Native Americans and non-Hispanic whites remained for 4 of the 5 measurements even after multivariable adjustment. In addition, multivariable adjustment did not account for the differences between Asians and Hispanics in the 14 or more activity-limited days group or for Hispanics with fair/poor health status. The differences in these measurements may be related to severity of disease, comorbidities, or disparities in treatment and access to care (16-18). Cultural and other differences in reporting may also be a factor (19,20). For example, Native Americans may have more disability and comorbid chronic conditions than other groups, which may negatively affect their HRQOL (20). Further evaluation with culturally appropriate techniques may better characterize HRQOL among individual racial/ethnic groups.

As people age and develop disease, they would be expected to report lower HRQOL than younger people. In our study, adults aged 65 years or older were less likely to report 14 or more mentally unhealthy days but more likely to report fair/poor health status and 14 or more physically unhealthy days compared with those aged 18 to 34 years. The finding that mentally unhealthy days was not different between the youngest age group and those aged 35 to 49 years and those aged 50 to 64 years but was higher compared with the oldest age group was unexpected. At least 3 explanations could account for these differences. First, the stigma associated with mental illness may lead to underreporting of days when mental health was not good, particularly among older people (21). Second, older people may adjust to their disease limitations by developing successful coping strategies, and, therefore, feel less compelled to report limitations related to health (22). Third, people with more severe disease or lower HRQOL or both may die earlier and not survive to 65 years of age. Additional research in HRQOL, including evaluation of link between employment and quality of life, could further characterize reasons for these differences.

In our study, women reported similar prevalence estimates of unhealthy days compared with men. However, multivariable analyses showed that the prevalence of 4 of the 5 unhealthy days measurements among women was significantly higher than the prevalence among men after accounting for differences related to age, race/ethnicity, education, income, household size, and health insurance coverage. The measure of fair/poor health status was not different from men in the multivariable analysis. Our study is consistent with other studies that demonstrate that women frequently report lower HRQOL than men (7,11,12,23). Although CHD is common in both men and women, women may manifest different symptoms, be diagnosed later in the course of the disease, and report a lower quality of life than men (3,11,15). A study in Hispanic patients with CHD identified higher rates of poor quality of life in women and suggested low social support and isolation as contributors to the higher rate in women (11).

We demonstrated that lower levels of educational attainment and income were both significantly related to the likelihood of lower HRQOL among people with CHD. Low levels of education and low income are generally associated with heart disease risk factors and poor clinical outcomes (15). People with higher levels of education may be exposed to more health messages, have better social support, be more aware of the importance of maintaining health, and be less likely to suffer from disease complications because they have more timely access to health care. Associations of higher HRQOL with higher incomes among people with CHD could be related to the ability to pay for more healthful foods and to obtain earlier and better quality health care that may decrease the severity of CHD through better control of the disease.

These findings are subject to at least 4 limitations. First, BRFSS is based on self-reported information and is subject to recall bias that may either overestimate or underestimate CHD. However, reported CHD is valid and reliable when self-reported data are compared with other sources such as medical record review and in-person interviews (24-27). Second, BRFSS does not survey CHD patients living in nursing homes or long-term–care facilities who likely would have more functional limitations and would be expected to report lower HRQOL than those living in the community. Third, this study does not examine treatment of or severity of CHD, which would be informative because HRQOL is likely related to the duration and severity of disease. Last, this study is cross-sectional and does not allow an assessment of the relationship between HRQOL and CHD over time. Indeed, some patients may alter their lifestyle after a diagnosis of CHD and improve their quality of life.

The BRFSS data also have strengths, such as being representative of community-dwelling adults in the United States. The estimates of self-reported CHD in BRFSS are comparable to those seen in other national surveys using self-reported information (28). The estimates of CHD among the socioeconomic groups in this study are similar to those reported previously with BRFSS data (12). Data from BRFSS give reliable and valid results across many measurements, including those associated with the survey's HRQOL measurements (13,25,29,30).

Early detection, education, and appropriate referrals for proper care are important tools for public health, clinical, and other health professionals to help reduce the overall burden of CHD. It is important, particularly in people with CHD, to encourage healthful lifestyles and adherence to treatment plans to minimize progression of disease. In community-dwelling adults with CHD, there are disparities in HRQOL among socioeconomic groups, especially for Native Americans, women, and older people. Tailoring interventions to people with CHD and poor HRQOL may assist in the overall management of CHD.

## Figures and Tables

**Table 1:** Prevalence of Self-Reported Coronary Heart Disease Among Adults Aged 18 Years or Older, 2007 Behavioral Risk Factor Surveillance System^a^

**Characteristic**	n	% (95% CI)
**Age group, y**		
18-34	56,420	1.1 (0.9-1.3)
35-49	106,282	2.7 (2.5-2.9)
50-64	129,541	8.3 (8.0-8.6)
≥65	113,398	19.1 (18.7-19.6)
**Sex**		
Female	251,832	4.7 (4.6-4.9)
Male	153,809	7.8 (7.6-8.0)
**Race/ethnicity**		
Non-Hispanic white	321,505	5.9 (5.8-6.0)
Non-Hispanic black	30,739	6.0 (5.6-6.5)
Hispanic	29,223	6.8 (6.2-7.5)
Asian	6,075	2.6 (2.0-3.4)
Native American^b^	5,683	11.5 (9.9-13.4)
Other	12,394	7.5 (6.6-8.4)
**Education**		
<12 y	39,697	9.6 (9.0-10.2)
12 y or equivalent	123,323	6.7 (6.5-7.0)
Some college	107,047	6.3 (6.0-6.6)
College graduate	134,669	4.8 (4.6-5.0)
**Annual household income, $**		
<20,000	63,735	10.3 (9.8-10.8)
20,000-34,999	78,767	7.1 (6.8-7.5)
35,000-49,999	58,233	5.9 (5.5-6.3)
≥50,000	153,511	4.9 (4.6-5.2)
Unknown/refused	51,109	5.7 (5.3-6.1)
**Household size**		
1	113,492	6.7 (6.4-7.0)
2	148,105	6.3 (6.1-6.5)
≥3	143,922	5.8 (5.5-6.2)
**Health insurance coverage**		
Yes	357,956	5.9 (5.8-6.1)
No	46,801	6.3 (5.8-6.9)
Unknown/refused	884	4.6 (2.9-7.2)
**Overall**	405,641	6.1 (6.0-6.3)

Abbreviation: CI, confidence interval.

a All estimates are weighted and age-standardized to the 2000 US standard population, except for age-specific estimates.

b Respondents who self-identified as American Indian or Alaska Native.

**Table 2a:** Self-Reported Health Status and Unhealthy Days During the Previous 30 Days Among Adults With Self-Reported Coronary Heart Disease, 2007 Behavioral Risk Factor Surveillance System

Characteristic	n^a^	Health Status Fair/Poor		≥14 PhysicallyUnhealthy Days		≥14 Mentally Unhealthy Days	
		%^b^ (95% CI)^c^	*P* Value^d^	%^b^ (95% CI)^c^	*P* Value^d^	%^b^ (95% CI)^c^	*P* Value^d^
**Age group, y**							
18-34	523	37.3 (28.9-46.6)	[Reference]	17.8 (12.0-25.5)	[Reference]	26.0 (18.4-35.3)	[Reference]
35-49	2,810	51.8 (48.1-55.4)	.003	36.1 (32.7-39.6)	<.001	30.3 (27.1-33.7)	.35
50-64	10,853	52.6 (50.8-54.4)	.001	37.1 (35.3-38.9)	<.001	22.7 (21.1-24.4)	.46
≥65	21,192	48.4 (47.1-49.8)	.02	29.5 (28.4-30.7)	<.001	10.0 (9.2-10.9)	<.001
**Sex**							
Female	17,718	49.2 (45.2-53.2)	.21	32.1 (29.6-34.6)	.06	25.0 (22.2-27.9)	.56
Male	17,660	45.3 (41.0-49.8)	[Reference]	27.7 (24.1-31.6)	[Reference]	23.4 (19.4-28.0)	[Reference]
**Race/ethnicity**							
Non-Hispanic white	28,939	42.4 (39.5-45.4)	[Reference]	29.8 (27.1-32.6)	[Reference]	24.7 (21.8-27.9)	[Reference]
Non-Hispanic black	2,287	46.0 (39.8-52.2)	.31	27.6 (23.6-32.1)	.40	28.9 (21.6-37.7)	.34
Hispanic	2,068	59.8 (53.9-65.4)	<.001	30.4 (25.4-35.9)	.84	25.4 (21.6-37.7)	.85
Asian	238	20.6 (13.3-30.6)	<.001	17.8 (8.6-33.3)	.06	3.9 (1.6-8.9)	<.001
Native American^e^	631	65.7 (51.4-77.6)	<.001	50.4 (36.9-63.7)	.004	30.2 (20.6-42.0)	.34
Other	1,213	42.5 (32.9-52.6)	.99	34.9 (25.9-45.2)	.32	24.9 (16.9-35.1)	.97
**Education**							
<12 y	6,107	68.3 (61.7-74.2)	<.001	40.5 (34.5-46.7)	<.001	32.8 (26.4-39.9)	<.001
12 y or equivalent	12,292	47.2 (43.5-51.0)	<.001	28.7 (26.1-31.5)	<.001	21.8 (18.9 -25.1)	.002
Some college	9,031	41.6 (36.3-47.0)	<.001	30.2 (26.4-34.2)	<.001	25.7 (21.3-30.6)	<.001
College graduate	7,851	27.9 (24.3-31.9)	[Reference]	17.9 (15.5-20.5)	[Reference]	13.5 (9.7-18.5)	[Reference]
**Annual household income, $**							
<20,000	9,920	66.4 (60.4-71.9)	<.001	45.2 (40.4-50.2)	<.001	35.6 (30.0-41.5)	<.001
20,000-34,999	8,598	50.2 (45.0-55.4)	<.001	31.0 (26.9-35.5)	<.001	24.6 (20.5-29.1)	<.001
35,000-49,999	4,443	34.6 (29.9-39.5)	<.001	20.2 (16.9-23.9)	.003	18.0 (13.3-23.9)	.22
≥50,000	7,267	21.9 (20.7-30.2)	[Reference]	13.9 (11.6-16.4)	[Reference]	13.6 (9.6-18.8)	[Reference]
Unknown/ refused	5,127	52.2 (45.9-58.4)	<.001	30.4 (25.6-35.7)	<.001	19.7 (15.9-24.1)	.05
**Household size**							
1	14,649	46.0 (39.3-52.8)	.69	34.5 (28.2-41.3)	.11	30.4 (22.0-40.4)	.21
2	15,252	49.2 (44.1-54.4)	.61	32.0 (28.1-36.2)	.18	23.6 (19.4-28.3)	.86
≥3	5,465	47.6 (43.8-51.4)	[Reference]	28.6 (25.6-31.7)	[Reference]	24.1 (20.8-27.8)	[Reference]
**Health insurance coverage**							
Yes	32,753	43.7 (40.3-47.1)	[Reference]	28.6 (26.3-31.1)	[Reference]	22.4 (19.7-25.3)	[Reference]
No	2,566	55.5 (50.0-60.9)	<.001	33.0 (28.3-38.0)	.12	27.4 (22.4-33.0)	.10
**Overall**	35,378	46.9 (43.8-49.9)	NA	29.5 (27.1-31.9)	NA	23.9 (21.2-26.9)	NA

Abbreviations: CIs, confidence interval; NA, not applicable.

a Unweighted total sample size. Some categories do not total 35,378 because of missing data.

b Weighted prevalence estimates, except for age groups, are age-standardized to the 2000 US standard population.

c CI around the weighted prevalence estimate.

d Significant differences were defined as *P* < .05 based on pairwise comparisons to reference group for each characteristic.

e Respondents who self-identified as American Indian or Alaska Native.

**Table 2b:** Self-Reported Unhealthy Days and Activity-Limited Days During the Previous 30 Days Among Adults With Coronary Heart Disease, 2007 Behavioral Risk Factor Surveillance System

Characteristic	n^a^	≥14 Total Unhealthy (Physical or Mental) Days		≥14 Activity-Limited Days	
		%^b^ (95% CI)^c^	*P* Value^d^	%^b^ (95% CI)^c^	*P* Value^d^
**Age group, y**					
18-34	523	34.0 (26.0-43.0)	[Reference]	13.3 (8.3-20.5)	[Reference]
35-49	2,810	49.7 (46.0-53.3)	.001	27.9 (25.0-30.9)	<.001
50-64	10,853	44.3 (42.4-46.1)	.02	25.6 (24.0-27.2)	<.001
≥65	21,192	33.9 (32.7-35.2)	.98	16.6 (15.6-17.6)	.29
**Sex**					
Female	17,718	44.2 (40.9-47.7)	.06	22.8 (20.6-25.1)	.14
Male	17,660	38.9 (34.6-43.3)	[Reference]	19.7 (16.6-23.3)	[Reference]
**Race/ethnicity**					
Non-Hispanic white	28,939	42.5 (39.2-45.7)	[Reference]	23.4 (20.8-26.2)	[Reference]
Non-Hispanic black	2,287	46.8 (38.8-54.9)	.33	20.8 (16.2-26.3)	.36
Hispanic	2,068	41.8 (35.9-48.0)	.86	16.2 (12.5-20.7)	.004
Asian	238	20.3 (10.6-35.4)	<.001	4.0 (2.1-7.5)	<.001
Native American^e^	631	57.5 (43.7-70.1)	.03	45.8 (33.1-59.2)	.001
Other	1,213	43.5 (33.3-54.3)	.86	26.7 (18.6-36.8)	.49
**Education**					
<12 y	6,107	52.0 (45.3-58.7)	<.001	26.8 (21.8-32.5)	<.001
12 y or equivalent	12,292	40.9 (37.1-44.7)	<.001	21.8 (19.3-24.6)	.001
Some college	9,031	43.2 (38.2-48.4)	.001	21.2 (18.0-24.7)	.005
College graduate	7,851	25.8 (21.4-30.7)	[Reference]	13.7 (10.1-18.3)	[Reference]
**Annual household income, $**					
<20,000	9,920	58.1 (52.3-63.7)	<.001	33.3 (29.0-37.8)	<.001
20,000-34,999	8,598	41.7 (36.9-46.7)	<.001	22.4 (18.7-26.5)	<.001
35,000-49,999	4,443	30.0 (25.0-35.5)	.18	12.6 (9.8-16.1)	.23
≥50,000	7,267	25.1 (20.7-30.2)	[Reference]	9.8 (7.0-13.6)	[Reference]
Unknown/refused	5,127	41.8 (35.8-48.1)	<.001	21.8 (17.5-26.8)	<.001
**Household size**					
1	14,649	46.3 (37.5-55.5)	.24	25.7 (19.9-32.6)	.08
2	15,252	43.8 (38.9-48.9)	.29	25.4 (21.5 -29.8)	.02
≥3	5,465	40.5 (36.9-44.2)	[Reference]	19.6 (17.1-22.4)	[Reference]
**Health insurance coverage**					
Yes	32,753	38.8 (35.9-41.8)	[Reference]	20.6 (18.5-22.8)	[Reference]
No	2,566	45.7 (40.2-51.3)	.03	23.5 (19.5-28.0)	.23
**Overall**	35,378	41.0 (38.1-44.0)	NA	20.9 (18.9-23.1)	NA

Abbreviations: CI, confidence interval; NA, not applicable.

a Unweighted total sample size. Some categories do not total 35,378 because of missing data.

b Weighted prevalence estimates, except for age groups, are age-standardized to the 2000 US standard population.

c CI around the weighted prevalence estimate.

d Significant differences were defined as *P *< .05 based on pairwise comparisons to referent group for each characteristic.

e Respondents who self-identified as American Indian or Alaska Native.

**Table 3a:** Adjusted Odds Ratio^a^ (AOR) for Fair/Poor Health Status, Physically Unhealthy Days, and Mentally Unhealthy Days During the Previous 30 Days Among Adults With Self-Reported Coronary Heart Disease, 2007 Behavioral Risk Factor Surveillance System

Characteristic	Health Status Fair/Poor		≥14 Physically Unhealthy Days		≥14 Mentally Unhealthy Days	
	AOR (95% CI)	*P* Value^b^	AOR (95% CI)	*P* Value^b^	AOR (95% CI)	*P* Value^b^
**Age group, y**						
18-34	1 [Reference]	[Reference]	1 [Reference]	[Reference]	1 [Reference]	[Reference]
35-49	3.2 (2.2-4.9)	<.001	3.3 (2.0-5.4)	<.001	1.6 (1.0-2.7)	.04
50-64	4.2 (2.8-6.2)	<.001	3.8 (2.4-6.2)	<.001	1.3 (0.8-2.0)	.35
≥65	3.1 (2.1-4.7)	<.001	2.3 (1.4-3.7)	<.001	0.4 (0.3-0.7)	<.001
**Sex**						
Female	1.1 (1.0-1.2)	.10	1.2 (1.1-1.4)	<.001	1.3 (1.1-1.5)	<.001
Male	1 [Reference]	[Reference]	1 [Reference]	[Reference]	1 [Reference]	[Reference]
**Race/ethnicity**						
Non-Hispanic white	1 [Reference]	[Reference]	1 [Reference]	[Reference]	1 [Reference]	[Reference]
Non-Hispanic black	1.2 (1.0-1.5)	.02	0.9 (0.8-1.1)	.22	0.8 (0.7-1.0)	.09
Hispanic	1.5 (1.2-1.9)	<.001	0.8 (0.6-1.0)	.04	0.8 (0.6-1.1)	.24
Asian	0.6 (0.3-1.2)	.14	0.5 (0.2-1.2)	.12	0.3 (0.1-1.3)	.11
Native American^c^	1.7 (1.1-2.5)	.57	1.7 (1.1-2.5)	.01	1.0 (0.7-1.5)	.81
Other	1.1 (0.8-1.4)	.01	1.1 (0.9-1.5)	.40	1.2 (0.8-1.6)	.37
**Education**						
<12 y	2.5 (2.1-2.9)	<.001	1.7 (1.4-2.0)	<.001	1.8 (1.4-2.3)	<.001
12 y or equivalent	1.5 (1.3-1.6)	<.001	1.2 (1.0-1.3)	.02	1.2 (1.0-1.5)	.05
Some college	1.3 (1.1-1.5)	<.001	1.2 (1.0-1.4)	.01	1.4 (1.1-1.7)	.001
College graduate	1 [Reference]	[Reference]	1 [Reference]	[Reference]	1 [Reference]	[Reference]
**Annual household income, $**						
<20,000	4.3 (3.7-5.0)	<.001	4.2 (3.6-4.9)	<.001	4.0 (3.2-4.9)	<.001
20,000-34,999	2.4 (2.1-2.8)	<.001	2.5 (2.1-2.9)	<.001	2.4 (1.9-3.0)	<.001
35,000-49,999	1.6 (1.4-1.9)	<.001	1.5 (1.3-1.8)	<.001	1.7 (1.3-2.2)	<.001
≥50,000	1 [Reference]	[Reference]	1 [Reference]	[Reference]	1 [Reference]	[Reference]
Unknown/ refused	2.2 (1.9-2.6)	<.001	2.4 (2.0-2.8)	<.001	2.3 (1.8-3.0)	<.001
**Household size**						
1	0.8 (0.7-1.0)	.01	0.9 (0.8-1.1)	.22	0.8 (0.7-1.0)	.06
2	1.0 (0.9-1.1)	.97	1.0 (0.9-1.2)	.91	0.9 (0.7-1.0)	.07
≥3	1 [Reference]	[Reference]	1 [Reference]	[Reference]	1 [Reference]	[Reference]
**Health insurance coverage**						
Yes	1 [Reference]	[Reference]	1 [Reference]	[Reference]	1 [Reference]	[Reference]
No	1.1 (0.9-1.3)	.40	1.0 (0.8-1.2)	.71	1.1 (0.9-1.4)	.51

Abbreviation: CI, confidence interval.

a Reflects adjustment for all other variables listed in the table with appropriate reference group listed.

b Calculated by using *t* test for beta coefficients.

c Respondents who self-identified as American Indian or Alaska Native.

**Table 3b:** Adjusted Odds Ratio^a^ (AOR) for Unhealthy Days and Activity-Limited Days During the Previous 30 Days Among Adults With Self-Reported Coronary Heart Disease, 2007 Behavioral Risk Factor Surveillance System

Characteristic	≥14 Total Unhealthy (Physical or Mental) Days		≥14 Activity-Limited Days	
	AOR (95% CI)	*P* Value^b^	AOR (95% CI)	*P* Value^b^
**Age group, y**				
18-34	1 [Reference]	[Reference]	1 [Reference]	[Reference]
35-49	2.5 (1.6-3.8)	<.001	2.9 (1.7-5.0)	<.001
50-64	2.2 (1.5-3.4)	<.001	2.7 (1.6-4.8)	<.001
≥65	1.2 (0.8-1.8)	.35	1.4 (0.8-2.4)	.28
**Sex**				
Female	1.4 (1.2-1.5)	<.001	1.2 (1.1-1.3)	.01
Male	1 [Reference]	[Reference]	1 [Reference]	[Reference]
**Race/ethnicity**				
Non-Hispanic white	1 [Reference]	[Reference]	1 [Reference]	[Reference]
Non-Hispanic black	1.0 (0.8-1.1)	.63	0.9 (0.7-1.1)	.22
Hispanic	0.8 (0.6-1.0)	.04	0.5 (0.4-0.7)	<.001
Asian	0.5 (0.3-1.1)	.09	0.2 (0.1-0.4)	<.001
Native American^c^	1.6 (1.1-2.3)	.02	1.9 (1.3-2.9)	.002
Other	1.1 (0.8-1.4)	.65	1.2 (0.9-1.6)	.28
**Education**				
<12 y	1.8 (1.5-2.1)	<.001	1.5 (1.2-1.8)	<.001
12 y or equivalent	1.3 (1.1-1.5)	<.001	1.1 (1.0-1.3)	.09
Some college	1.3 (1.1-1.5)	<.001	1.2 (1.0-1.4)	.06
College graduate	1 [Reference]	[Reference]	1 [Reference]	[Reference]
**Annual household income, $**				
<20,000	3.9 (3.3-4.5)	<.001	4.7 (3.9-5.7)	<.001
20,000-34,999	2.2 (1.9-2.5)	<.001	2.6 (2.1-3.1)	<.001
35,000-49,999	1.4 (1.2-1.6)	<.001	1.5 (1.2-1.9)	<.001
≥50,000	1 [Reference]	[Reference]	1 [Reference]	[Reference]
Unknown/refused	2.1 (1.8-2.5)	<.001	2.5 (2.1-3.1)	<.001
**Household size**				
1	0.9 (0.8-1.0)	.15	0.9 (0.7-1.0)	.048
2	1.0 (0.9-1.1)	.76	1.0 (0.9-1.2)	.81
≥3	1 [Reference]	[Reference]	1 [Reference]	[Reference]
**Health insurance coverage**				
Yes	1 [Reference]	[Reference]	1 [Reference]	[Reference]
No	1.0 (0.8-1.3)	.78	1.0 (0.8-1.3)	.78

Abbreviation: CI, confidence interval.

a Reflects adjustment for all other variables listed in the table with appropriate reference group listed.

b Calculated by using *t* test for beta coefficients.

c Respondents who self-identified as American Indian or Alaska Native.
